# The Limits of Predictive Remapping of Attention Across Eye Movements

**DOI:** 10.3389/fpsyg.2019.01146

**Published:** 2019-05-24

**Authors:** Kiki Arkesteijn, Artem V. Belopolsky, Jeroen B. J. Smeets, Mieke Donk

**Affiliations:** ^1^Department of Experimental and Applied Psychology, Vrije Universiteit Amsterdam, Amsterdam, Netherlands; ^2^Department of Human Movement Sciences, Vrije Universiteit Amsterdam, Amsterdam, Netherlands

**Keywords:** saccades, planning, sequence, behavior, replication study

## Abstract

With every eye movement, visual input projected onto our retina changes drastically. The fundamental question of how we keep track of relevant objects and movement targets has puzzled scientists for more than a century. Recent advances suggested that this can be accomplished through the process of predictive remapping of visual attention to the future post-saccadic locations of relevant objects. Evidence for the existence of predictive remapping of attention was first provided by Rolfs et al. (2011) (*Nature Neuroscience, 14*, 252–256). However, they used a single distant control location away from the task-relevant locations, which could have biased the allocation of visual attention. In this study we used a similar experimental paradigm as Rolfs et al. (2011), but probed attention equally likely at all possible locations. Our results showed that discrimination performance was higher at the remapped location than at a distant control location, but not compared to the other two control locations. A re-analysis of the results obtained by Rolfs et al. (2011) revealed a similar pattern. Together, these findings suggest that it is likely that previous reports of the predictive remapping of attention were due to a diffuse spread of attention to the task-relevant locations rather than to a specific shift toward the target’s future retinotopic location.

## Introduction

Our eye movement system has evolved to quickly bring the fovea – the area of the retina with the highest visual acuity – to the objects of interest. At the same time, with every eye movement, the visual input projected onto our retina changes dramatically. The fundamental question of how we keep track of relevant objects despite such fragmented and intermittent visual input has puzzled scientists over multiple decades. Several theories propose that visual attention preceding saccadic eye movements plays a crucial role in this process. While the premotor theory of attention (Rizzolatti et al., 1994), views visual attention as a by-product of eye movement programming, the visual attention model (Schneider, 1995; Schneider and Deubel, 2002), suggests that attention is necessary for accurate targeting of the eye movement system. Despite many variations on these views (Belopolsky and Theeuwes, 2009, 2012; Smith and Schenk, 2012), all theories agree that attention precedes saccades in most everyday situations. Interestingly, studies have also demonstrated that when saccades are planned, visual attention is narrowly allocated to the impending saccade goals. This is not only true for single saccades (Hoffman and Subramaniam, 1995; Kowler et al., 1995; Deubel and Schneider, 1996) but also for sequences of saccades (Godijn and Theeuwes, 2003; Baldauf and Deubel, 2008).

Given that the eye movement system operates in retinotopic coordinates, in order to successfully complete a saccade sequence, target locations need to be updated with each intervening saccade. Evidence for this updating of the visual scene has been observed by Jonikaitis and Belopolsky (2014), who showed that the saccade plan for the second saccade was based on information that was presented before the start sequence and therefore is “remapped” across the first saccade (for similar results see: Boon et al., 2018; Van Leeuwen and Belopolsky, 2018).

Neurophysiological recordings have identified predictive remapping of receptive fields – a mechanism that might underlie the updating of a visual scene. It was demonstrated that receptive fields of neurons in the brain areas responsible for spatial attention in monkeys predictively shift to the future post-saccadic location of an onset stimulus in anticipation of an eye movement (Duhamel et al., 1992; Walker et al., 1995; Yao et al., 2016). However, more recently a debate has arisen whether preceding an eye movement, receptive fields truly shift toward their future post-saccadic location (referred to as “remapped” location) or converge toward the saccade target (Tolias et al., 2001; Zirnsak and Moore, 2014; Zirnsak et al., 2014), or both (Neupane et al., 2016).

On the behavioral level, several studies have demonstrated anticipatory facilitation of visual processing at the location that would contain the target after the impending saccade, which was taken as a correlate of predictive remapping of receptive fields (Rolfs et al., 2011; Jonikaitis et al., 2013; Szinte et al., 2016, 2018). By pre-allocating attention to the retinotopic locations that would become relevant in the future (e.g., “attentional pointers”), we might be able to keep track of relevant objects despite intervening eye movements. It has also been suggested that such a mechanism can form the basis for the experience of visual stability (Cavanagh et al., 2010).

**FIGURE 1:**
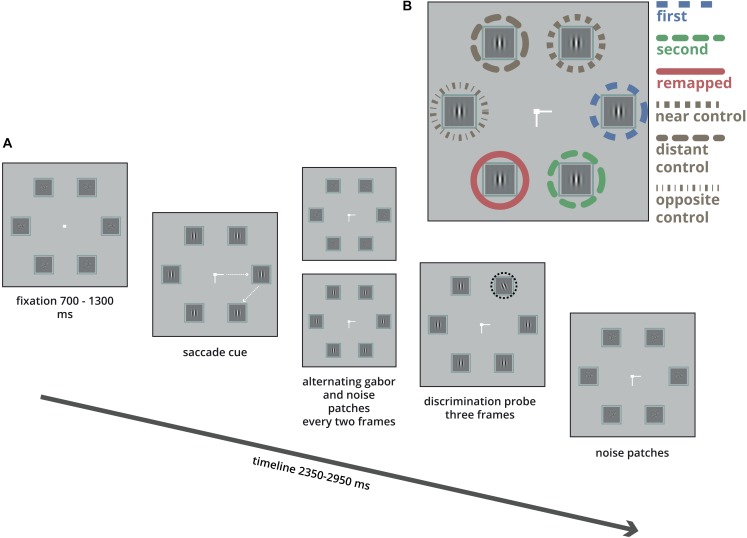
Illustration of a single trial. **(A)** Six boxes were arranged in a regular hexagon. Participants were instructed to make two saccades as quickly as possible, first to the left or right, (here to the right), and next up or down (here down). In each box Gabor patches and noise patches were alternating every two frames (17 ms). After a pre-specified time (see section “Materials and Methods”) one of the Gabor patches changed orientation (highlighted here in the black dashed circle) for 3 frames (25 ms), followed by noise patches. After the participants fixated the second target they indicated the orientation of the Gabor tilt using the right or left arrow key. **(B)** The tilted Gabor was equally likely presented in either one of the six boxes.

Given the large impact of these findings on our understanding of how visual attention is updated across saccades, it is important to establish the boundary conditions under which predictive remapping occurs. The aim of the present study was to investigate the role of voluntary bias in the allocation of attention on predictive remapping of attention before performing a saccade sequence. Specifically, in the original study investigating predictive remapping of attention by Rolfs et al. (2011), participants were instructed to perform two consecutive eye movements toward two neighboring target locations that were presented in a hexagon together with four other locations (see Figure 1). To examine the allocation of attention, observers had to indicate the orientation of a tilted Gabor grating (probe) which was briefly presented in close temporal proximity to the first saccade. Probes could be presented at either one of the two saccadic target locations (“first target” and “second target”), the “remapped” location, or a control location distant to these three locations (which we will refer to as the “distant control” location). Importantly, in their arrangement, the allocation of attention was not measured across all six possible locations but was limited to four locations. Moreover, the specific arrangement of the probe locations was such that the probe appeared in 75% of the trials at one of three neighboring locations (i.e., the two saccadic target locations and the remapped location) and in 25% of the trials at the distant control location. This set-up might have induced a voluntary bias in attention toward the neighboring locations at the expense of the distant control location. That is, in response to the central cue indicating the two consecutive saccadic targets, attention might have been coarsely allocated toward the three neighboring locations at which the probe was presented in 75% of all trials.

In order to investigate whether such a task-level manipulation of attention influenced the results on predictive remapping, we adopted the experimental paradigm of Rolfs et al. (2011) but probed attention at all six possible locations with an equal probability. Importantly, as opposed to Rolfs et al. (2011) who analyzed the time-course of discrimination performance at each location separately, we applied a conventional analysis of comparing the discrimination performance between specific locations (Posner, 1980). If predictive remapping of a saccade target results in a location-specific enhancement of attention at the future retinotopic location, we should find better discrimination performance at the remapped location than at the control locations and replicate the pattern of results of Rolfs et al. (2011) using a design where attention is probed at all possible locations. In addition to the saccade target locations and the remapped location, we test discrimination performance at three control locations: the one near to the first saccade target (“near control”), the distant control location (the one used in the main experiment by Rolfs et al., 2011), and the location opposite to the saccade direction (“opposite control”). Typically, studies on remapping of attention use highly trained participants who perform the task for several thousands of trials (Rolfs et al., 2011; Jonikaitis et al., 2013; Szinte et al., 2016). To examine the boundary conditions of this effect we have employed naïve participants and used an adaptive algorithm to set the probe onset for each trial so that it would be likely to occur just before the onset of the saccade. Furthermore, to get a precise estimate of discrimination performance as a function of probe presentation time relative to saccade onset we constructed a smoothed time-series for probe presentation times (Maij et al., 2010; Arkesteijn et al., 2018; Boon et al., 2018; Van Leeuwen and Belopolsky, 2018; Van Leeuwen et al., 2018). Under these boundary conditions, we also expected to find the typically reported parallel allocation of attention to both saccade targets (Godijn and Theeuwes, 2003; Baldauf and Deubel, 2008), which served as the benchmark for examining the predictive remapping of attention.

## Materials and Methods

### Participants

Forty-eight healthy university students (aged: 18–33, mean: 26, 28 women) of the Vrije Universiteit Amsterdam took part in the experiment. Pilot results showed that the dual task (double saccade and perceptual discrimination) was difficult for many participants. Therefore, we implemented a screening phase in which participants had to reach a threshold of discrimination performance in order to proceed to the experimental phase (criteria described in section 2.3: stimuli, design, and procedure). Thirty participants were excluded after the screening phase and did not proceed to the experimental phase. From the remaining 18 participants, eight of them were excluded from the analysis, because more than 45% of the trials in the experimental phase were rejected (criteria explained in section 2.4: Data Analysis). Ten participants (aged: 18–31, mean: 24, 7 women) were included in the data analysis. All had normal or corrected-to-normal vision and were naive to the purpose of the study. Informed consent was obtained from all participants and the experiment was approved by the Ethical Committee of the faculty of Behavioral and Movement Sciences of the Vrije Universiteit Amsterdam.

### Apparatus

The experiment was conducted in a dimly lit room. The stimuli were presented on a 21″ LCD monitor (Samsung 2233RZ) with a 1680 × 1050 pixel resolution and a 120 Hz refresh rate. Gaze was recorded using the Eyelink 1000 (SR research) with a temporal resolution of 1 ms and a spatial resolution of 0.01°. The experimental software controlling the stimulus presentation, response collection, and eye tracking was written with OpenSesame version 2.9 (Mathôt et al., 2012) using a PsychoPy back-end (Peirce, 2007) and PyGaze (Dalmaijer et al., 2014). An automatic algorithm detected saccades using minimum velocity and acceleration criteria of 35°/s and 9500°/s2.

### Stimuli, Design and Procedure

Participants were seated with their head positioned on a chin and forehead-rest at a distance of 70 cm from the display. Stimuli were presented on a gray (9 cd/m2) background. Each trial began with a white fixation square (107 cd/m2, 0.3°) presented at the center of the screen. The fixation square was surrounded by six 1.5° boxes evenly distributed in a hexagon with a radius of 5° (see Figure 1). Each box contained a stream of vertical Gabor patches (2.5 cycles per degree, with a random phase and maximum contrast) alternating every 17 ms with a white noise mask (randomly generated every trial). After a variable interval (Gaussian, μ = 1000 ms, σ = 300 ms) a central saccade cue was presented at the fixation square for 1650 ms. The saccade cue consisted of two lines that indicated the sequence of saccade target locations. One line was pointing left or right indicating the location of the first saccade target, this was always the one at the horizontal midline. Another line was pointing up or down indicating the location of the second saccade target, which was the one adjacent to the first target location either up or down (see Figure 1B). Participants were instructed to make a rapid sequence of two saccades toward these locations as soon as the lines appeared. There were four different saccade sequences (right-up, right-down, left-up, and left-down). Participants heard a short tone, (200 Hz, 100 ms) if they made a saccade in the wrong direction and received visual feedback when a saccade was executed too slowly (saccade latency above 450 ms).

Around the onset of the saccade (details in next paragraph), one of the six Gabor patches (the probe) was tilted left or right. The probe was presented for three frames (25 ms) while the other Gabor patches remained vertical for the same three frames. After this third frame, all boxes were filled with white noise for the rest of the trial. The probe could appear equally likely in all six boxes. Participants had to indicate whether the probe was tilted left or right using the corresponding arrow key immediately after executing the two consecutive saccades. A 2-up 1-down staircase procedure (with an angular step of 2°) was applied to obtain a 70% discrimination performance for the probe. A fraction correct of 0.5 would indicate performance at chance level whereas a fraction correct of 1.0 was a perfect score. For all participants, the tilt of the probe at the onset of the experiment was set at 22.5° from vertical. The minimum and maximum tilt were set at 3° and 45°, respectively. For instance, when the procedure would lead to a value below 3° (for example when the tilt was at 4.5° and a 2° angular step would produce a tilt of 2.5°) the next tilt was set at 3°. This procedure led to an average tilt of 11.2° from vertical (*SD* = 4.8).

Predictive remapping is thought to happen just before the onset of the saccade. Therefore, we were particularly interested in trials in which the probe would be presented about 50 ms before the start of saccade. To achieve this, the timing of the probe onset was set adaptively for each trial by subtracting 50 ms from the mean latency of the first saccade averaged over the preceding 10 valid trials. A trial was considered valid when the saccade latency was between 50 and 450 ms and the saccadic endpoint was within 2° of its target, which was determined online.

The experiment consisted of two 1-h sessions that took place on different days. The experiment consisted of blocks of 24 unique trials (i.e., 4 different saccade sequences × 6 different probe locations) which were presented in random order. The first session included a minimum of two and a maximum of six screening blocks. The orientation of the probe Gabor was fixed at 22.5° and was presented for 80 ms during the screening phase. Continuation to the experimental phase was dependent on performance: participants were only allowed to proceed when they could correctly discriminate at least 70% of all tilted probes and when more than 80% of their saccades in a block were directed at the two targets (when the eyes landed within 2° of both targets). The experimental phase consisted of at least 32 blocks resulting in a minimum of 768 and a maximum of 864 trials, this number was dependent on how many blocks could be completed within the 2 h of testing.

### Data Analysis

Eye-tracking data were analyzed offline using a custom written code (Python: Van Rossum and Drake, 2010) to extract all relevant details and events. The first saccade was defined as the first saccade that was initiated after the saccade cue was presented and landed within 2° of the first target. The second saccade was defined as the first saccade that followed the first saccade and ended within 2° of the second saccade target.

Trials were excluded when the first saccade latency was shorter than 80 ms or longer than 600, or when saccades did not land within 2° of the first target or 2° from the second target. Furthermore, trials were rejected when the probe-offset was before 125 ms preceding the first saccade or when the probe-offset was 125 ms after the saccade. For eight participants this resulted in a loss of more than 45% of the trials. These participants were excluded from further analysis. A total of ten participants and 5079 trials (66%) were included in the data analysis.

We made two sets of comparisons. For the first, we analyzed the data in a manner that follows the interpretation by Rolfs et al. (2011), neglecting the possibility that the control locations could differ. We therefore considered four types of probe locations: three special locations (first saccade target, second saccade target, and remapped location) and the “pooled control” location. For the latter, we pooled discrimination performance data that we obtained at the three control locations. To test our alternative explanation that the distant control location was special, we also compared performance at the remapped location with performance at each one of our three control locations separately.

We tested the time-course of these two sets of comparisons in two different ways. We used an analysis of time-bins in order to compare the results to the original study, and additionally, we used a more sensitive smoothed time series analysis.

**FIGURE 2:**
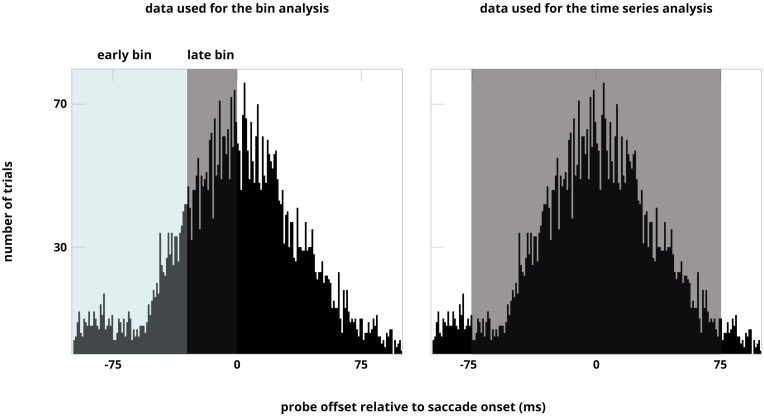
Distribution of the trials over all participants illustrated for both analyses. The distribution is ordered based on probe offset relative to saccade onset. Note that the sorting of data in time bins is based on number of trials.

For the time-bin analysis, we divided the trials into two time-bins based on probe-offset before saccade onset. We divided the time window -100 ms till 0 ms into two time bins based on a median split (see Figure 2). This resulted in two time bins, ranging from -100 ms till -25 ms (early), and -25 till 0 ms (late). The different time ranges for the two bins was due to a normal distribution of the trials that was centered around saccade onset. To assess whether there was a difference between discrimination performance for probes presented at the different locations over time we calculated a likelihood ratio to estimate whether performance at the locations differed from one another (the alternative hypothesis), or conversely, performance at the different locations was the same (null hypothesis). To do this Bayesian paired-samples *t*-tests between performance at the pooled control location, first, second, and remapped location for both time-bins (early and late) were calculated using default priors by JASP 0.9.0.1 (JASP Team, 2018). In addition, to assess whether there was a difference between discrimination performance for probes presented at the remapped location and the three different types of control locations over time, we ran a Bayesian paired-samples *t*-test between performance at the remapped location and the near, distant and opposite control locations for both time-bins (early and late). Bayes factors in favor of the null hypotheses were reported when BF01 > 3 and Bayes factor in favor of the alternative hypotheses were reported when BF10 > 3.

To get a precise estimate of discrimination performance as a function of probe presentation time relative to saccade onset for each participant and probe location we constructed a weighted smoothed time series for probe-offset times ranging from -75 ms till 75 ms (Maij et al., 2010; Arkesteijn et al., 2018; Boon et al., 2018; Van Leeuwen and Belopolsky, 2018; Van Leeuwen et al., 2018) using a moving Gaussian window (σ = 15 ms). This interval was chosen because of the majority of trials were centered around the onset of the saccade. To determine whether attention was predictively shifted toward the first and second saccade targets, as well as to the remapped location, discrimination performance at these locations was compared to performance at the pooled control location using a weighted paired sample *t*-tests for each sample of the smoothed time series. Similarly, the discrimination time-course at the remapped location was also compared to that at the three control locations separately.

We considered whether performance was significantly different when the *t*-test for a cluster of two or more consecutive time points had a *p*-value of <0.05. We controlled for multiple comparisons by cluster-based permutation testing: for every comparison (for instance: first target location vs pooled control location) the data was randomly assigned to either probe location and from this, a smoothed time series was constructed. This was done a thousand times for each participant. For each permutation, the sum of *t*-values for the largest cluster was used to construct a permutation distribution. We compared the sum of cluster *t*-values for the non-permuted data with the constructed distribution. The performance in a cluster was considered significantly different between two locations if the sum of cluster *t*-values for the non-permuted data fell outside the 95-percentile of the permuted distribution.

## Results

**FIGURE 3:**
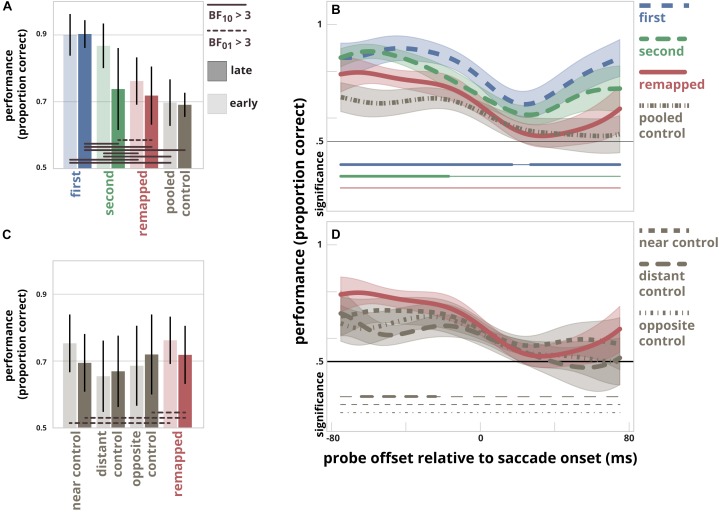
**(A)** Discrimination performance separately for the first saccade target location, the second saccade target location, the *remapped* location, and the *pooled control* location. The results are divided in two time-bins (mean times relative to saccade onset for the early and late bins were –49 ms and –11 ms, respectively). Error bars represent within-subject 95% confidence intervals. Solid horizontal lines connect pairs of probe locations for which it is likely that performance differed for the same time-bin, BF_10_ > 3. Striped lines connect pairs of probe locations for which it is likely that performance did not differ for the same time-bin, BF_01_ > 3. **(B)** The time-course of discrimination performance in relation to the probe presentation time relative to saccade onset for the first saccade target location, the second saccade target location, the *remapped* location, and the *pooled control* location. The curves depict the Gaussian (σ = 15 ms) smoothed time-series of discrimination performance at all four locations. The opaque areas indicate within-subject 95% confidence intervals. The clusters for which performance at the *pooled control* location differs significantly from that for the *first target* location, the *second target* location and the *remapped* location are indicated by thick horizontal lines below; thin line segments indicate a lack of significant difference. **(C)** Discrimination performance at the *remapped* location, and control location 1 to 3. Control location 2 was used by Rolfs et al. (2011). Format as in panel **A**. **(D)** The Gaussian smoothed time-series of discrimination performance at the *remapped* location and the three control locations. Format as in panel **B**.

The mean latency of the early time bin was -49 ms (*SD* = 21 ms), the mean latency of the late time bin was -11 ms (*SD* = 7 ms), see Figure 2. The latency of the first saccade was on average 267 ms (*SD* = 33 ms). Figure 3A shows discrimination performance at all locations divided into two time-bins. An estimated Bayes factor (null/alternative) between performance at the pooled control location, first target location, second target location, and remapped location for the early time-bin suggested that performance likely differed between first target location and pooled control location (BF10 = 26), first target location and remapped location (BF10 = 3.22), second target location and remapped location (BF10 = 3.96), and second target location and pooled control locations (BF10 = 82). The same analysis for the late time-bin revealed that performance likely differed between first target location and second target location (BF10 = 3.68), first target and remapped location (BF10 = 9), and first target location and pooled control location (BF10 = 381). In contrast, an estimated Bayes factor computed for the late time-bin suggested that it was more likely that performance did not differ between the second target location and remapped location (BF01 = 3.08).

Figure 3B shows time-series of discrimination performance at all locations as a function of probe presentation time relative to saccade onset. There was a difference between performance measured at the first target location compared to performance at the pooled control location for the time-points -75 ms till 17 ms and time-points 27 ms till 75 ms. Likewise, there was a difference between performance measured at the second target location compared to performance at the pooled control location for the time-points -75 ms till -17 ms. We found no differences in performance between the remapped location and the pooled control location at any time point.

In order to determine whether the lack of difference between performance at the remapped location and the control locations was dependent on the choice of control location, we compared the performance at these four locations (Figure 3C,D). An estimated Bayes factor (null/alternative) suggested that performance did not differ for all comparisons made. In contrast, an estimated Bayes factor computed for the early time-bin suggested that it was more likely that performance did not differ between the near control location and remapped location (BF01 = 3.21). Furthermore, the same analysis for the late time-bin revealed that it was more likely that performance did not differ between the near control location and remapped location, and between the opposite control location and the remapped location for the late time-bin (BF01 = 3.04 and BF01 = 3.24, respectively) (Figure 3C).

Figure 3D shows the smoothed time-series for the three different control locations and the remapped location. Cluster-based permutation testing revealed a difference between performance measured at the remapped location compared to the distant control location [location also used in the main experiment by Rolfs et al. (2011)] for the time-points between 64 and 24 ms before saccade onset. There were no significant differences found for every other comparison that was made.

## Discussion

The aim of our study was to investigate the occurrence of predictive remapping of attention prior to a sequence of two saccades while probing attention equally often at all possible stimulus locations. We used naïve participants and a novel adaptive algorithm to estimate the probe presentation times. The present results replicate previous studies demonstrating parallel deployment of attention to several locations before executing a saccade sequence (Godijn and Theeuwes, 2003; Baldauf and Deubel, 2008; Rolfs et al., 2011). Just as in these previous studies we observed enhanced discrimination performance for probes presented at the two saccade target locations relative to probes presented at any of the control locations prior to the execution of a saccade sequence.

Despite several differences in the experimental design, we replicated the original pattern of results of Rolfs et al. (2011): a small but significant increase in discrimination performance at the future post-saccadic location of the second saccade target relative to the distant control location (Figure 3B). Importantly, however, we found that discrimination performance did not differ between this remapped location and the near or opposite control locations. This was evident from both the time-bin analysis, as well as from the more sensitive time series analysis (Figure 3C,D, respectively). Furthermore, we found that discrimination performance was enhanced mostly at the two saccade targets and to a lesser degree at locations that neighbor the saccade targets: the near control location (dotted gray curve in Figure 3D) and the remapped location (red curve in Figure 3D). Although discrimination performance averaged across the late time bin did not differ between the second target location and the pooled control location (Figure 3A), performance actually differed between these locations up till 17 ms preceding the saccade, as was evident from the time series analysis in Figure 3B. Together, this pattern of results suggests that there is not a specific focus of attention at the remapped location, but rather a spread of attention around the two saccade target locations before the onset of the saccade.

The authors of the original study have also considered a spread of attention as an alternative explanation of the results of their main experiment and conducted two control experiments to test it. In these experiments, they used as their control location the near control instead of the distant control. The authors found an increase in discrimination performance over time at the remapped location, but not at the near control location, which was taken as the evidence against a spread of attention. However, Rolfs et al. (2011) never directly compared discrimination performance between remapped and control locations.

**FIGURE 4:**
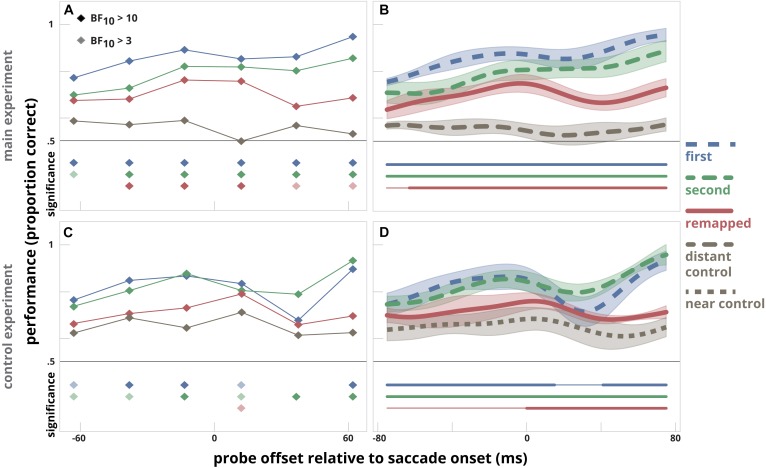
The data of Rolfs et al. (2011) reanalyzed in a similar way as our data was analyzed in Figure 3. Data depicted are derived from their main experiment **(A,B)** and their control experiment **(C,D)**. The left panels **(A,C)** show the time-course indicated by the time-bin analysis. The same bin sizes are used as in the main experiment by Rolfs et al. (2011). To assess whether there was a difference between discrimination performance at the *first target, second target*, and *remapped* location versus the control location (*distant control* in the main experiment and *near control* location in the control experiment), a likelihood ratio was calculated using a Bayesian paired sample *t*-test. The filled diamonds below the time bins indicate when there is strong evidence (BF_10_ > 10) that discrimination performance at that bin is higher than measured at the control location, an opaque diamond indicates when there is moderate evidence (BF_10_ > 3, BF_10_ < 10) that discrimination performance at that bin is higher than measured at the control location. The right panels **(B,D)** indicate the time-course of discrimination performance in relation to the probe presentation time relative to saccade onset for the *first* and *second* saccade target location, the *remapped* location and either the *distant control* location (main experiment) or the *near control* location (control experiment). The clusters for which performance at the *first target* location, the *second target* location, and the *remapped* location differ significantly from that at the control location are indicated by thick horizontal lines below; thin line segments indicate a lack of significant difference.

To directly compare probe discrimination performance at different locations, we subjected the original data of Rolfs et al. (2011), made available by the authors, to the same analyses that we used for our experiments. We plotted the results of their main experiment and first control experiment in Figure 4. Our re-analysis of the results they obtained in the main experiment revealed that discrimination performance was higher at the remapped location than at the distant control location (Figure 4, Panels A and B), which is similar to our results (Figure 3D). The re-analysis of the first control experiment revealed that discrimination performance at the remapped location did not differ from that at the near control location prior to the saccade. This was the case for all time bins preceding the saccade (Figure 4, panel C) and also evident from the analyses on the smoothed time series (panel D). From this we conclude that results that were obtained in our experiment were similar to the results of the original experiment.

Rolfs et al. (2011) also executed a second control experiment that used the near control location but cued the saccade sequence in a different way. Here, the pattern of results differed: performance was slightly higher for the remapped location compared to the near control location for a period from 55 ms before the saccade until 45 ms after the saccade. We can only speculate why changing the way of cueing changed the deployment of attention. Combined with the results of the other experiments, we conclude that making a saccade sequence in itself does not necessarily results in predictive remapping of attention.

A surprising finding from our re-analysis of the data by Rolfs et al. (2011) is that the probe was discriminated just as well during a saccade as before or after a saccade at nearly all locations (Figure 4B,D). This is surprising, given the well-known profound suppression in visual motion processing during saccades (Burr et al., 1994, 1999; Ross et al., 1996), although motion of patterns with low spatial frequency can sometimes be sensed (Castet and Masson, 2000). As the pattern of results in Figure 4B,D is affected by the smoothing; we examined the (lack of) saccadic suppression more closely. We compared detection performance during the saccade in our experiment with that in Rolfs et al. (2011). We restricted our analysis to trials in which the probe was presented 20–30 ms after the saccade onset. In this way, the probe presentation occurred completely during the saccadic eye movement. We found that during the saccade, performance in our data dropped from 67 to 51% correct, close to the expected chance-level of 50%. However, in the main experiment of Rolfs et al. (2011), performance during the saccade remained at the same level as before the saccade: 72% correct. We have no explanation for the fact that discrimination performance was unaltered during saccades in the main experiment by Rolfs et al. (2011).

Overall, using a direct comparison between locations, the patterns of results obtained in our study and the study of Rolfs et al. (2011) show to be highly similar. In both studies we find a small but significant increase in discrimination performance at the remapped location compared to the distant control location and no difference in discrimination performance compared to the near control location. This pattern of results is not consistent with the predictive remapping of attention proposed in the original study.

When comparing discrimination performance at the remapped location and the distant control location, we found only a small effect, while the effect in the original study appeared to be larger. This may have to do with more focused allocation of attention in the study of Rolfs et al. (2011), which was induced by the specific arrangement of probe locations in their study. Attention might have been voluntarily allocated toward the three neighboring locations at which the probe was presented in 75% of all trials at expense of the control location where the probe was presented only in 25% of all trials. However, it is important to note that our study is a conceptual replication, and differs from the study of Rolfs et al. (2011) in more aspects than the arrangement of probe locations. In our study, we used a different approach for determining the time to present the probe; such that it was dependent on saccade latency. Likewise, the probe presentation stream differed from Rolfs et al. (2011), in our design the stream alternating between Gabor and noise patches stopped after the presentation of the probe, however, in their study the alternation continued. Any differences found between our studies could be due to the variations implemented in our design.

Our results relate to earlier findings by Puntiroli et al. (2015), who tested the allocation of attention at targets and non-targets while making voluntary and involuntary saccades. In their experiment participants made either one or two saccades. In 73% of the trials, participants made a correct saccade toward the target, while ignoring a distractor. Here, they found no difference between discrimination performance at the remapped location versus the control location. In 27% of trials, participants made a saccade toward the distractor. In this case, the spatial layout resembled that of what was used by Rolfs et al. (2011). Likewise, there was initially no difference found between discrimination performance at the remapped location vs the control location. However, when the data was split based on inter-saccadic interval latencies they found a difference between performance at the remapped vs control locations for the fastest latencies. Our conclusions are different from Szinte and colleagues (Szinte et al., 2015, 2016, 2018), who reported performance differences between remapped and other locations just prior to a saccade. However, it is important to note that these latter studies were primarily concerned with changes in sensitivity for motion direction rendering any direct comparison with our results difficult.

The enhancement of attention at the future retinal location has been supported by several neurophysiological studies. On a neurophysiological level, neural activity is remapped to the future receptive field. Several areas that are involved with spatial attention such as the lateral intraparietal area (Duhamel et al., 1992), superior colliculus (Walker et al., 1995), and middle temporal area (Yao et al., 2016) show “forward” remapping of neural activity. Conversely, our finding that discrimination was enhanced mostly at the saccade target locations is in line with electrophysiological recordings performed in the frontal eye fields in monkeys (Zirnsak et al., 2014), which led to an alternative view on predictive remapping, namely that attention is deployed mostly at the future saccadic target before the onset of the eye movement instead of at the future retinal location and is therefore a “convergent” type of remapping (Zirnsak and Moore, 2014). Additionally, a spatial unspecific type of remapping has been found for neurons in V4, they exhibit a pre-saccadic attentional modulation without a shift in spatial tuning (Marino and Mazer, 2018). Together these studies show that attention shifts are differently represented by different brain areas.

The spread of attention we found resembles the spread that has been found in studies where attention was cued before the onset of a saccade (Harrison et al., 2012; Jonikaitis et al., 2013). In these studies, attention was spread to the cued “hemifield” of the display and was not confined to the specific locations tested. However, other studies did find a narrow distribution of attention at saccade targets and no deployment of attention at a location in-between saccade targets (Godijn and Theeuwes, 2003; Baldauf and Deubel, 2008). The discrepancies between different behavioral results could possibly be explained by differences in probe task difficulty. In our study participants reported that performing a combined perceptual discrimination task and a double-step saccade was extremely difficult. Kowler et al. (1995) have shown that the relative importance of saccade and probe task can affect the way attention is allocated before the execution of a single saccade. It is possible that in our study as well as in Rolfs et al. (2011), participants have eluded to adopting strategies such as allocating attention to the probe locations around the two saccade targets in order to achieve the desired level of discrimination performance. Future research should investigate the role of the difficulty of the probe task on the allocation of attention in the double-saccade task.

Recently, Szinte et al. (2018) found that attention was allocated to the remapped location before the onset of a saccade, given that the saccade cue was presented sufficiently early enough preceding the saccade. They used a performance discrimination task similar to the study of Rolfs et al. (2011) and our present study, but the amplitude of the saccades they used was twice as large and attention was probed at more locations. They found no spreading of attention and no attentional benefits at the locations between the fixation and the saccade target. Attention was measured to be higher at the saccade target, cued target and in a lesser degree at the remapped target. It is possible that attentional spreading depends on the size of the saccade, with more spreading occurring for smaller saccades, as in our study, and a narrower focus of attention for larger saccades, as in the study by Szinte et al. (2018). Furthermore, having a larger set of possible probe locations per trial, 12 vs 6, could lead to attention being allocated mostly at the saccade target with less spilling to nearby locations, as was the case in the study by Szinte et al. (2018).

To summarize, in the present study we determined the limits of observing predictive remapping of attention by performing a conceptual replication of the study by Rolfs et al. (2011). We demonstrated that while we were able to replicate the allocation of attention to two saccade targets prior to execution of saccade sequence, we did not find that attention was specifically enhanced at the future retinotopic location of the second target as reported by Rolfs et al. (2011). Furthermore, we performed a re-analysis of the original study, which confirmed our results. Overall, our results are in line with the idea of a spread of attention around the saccade target locations and suggest that participants might have adopted a strategy of prioritizing probe locations around the saccade targets to overcome the high dual-task demands.

## Author’s Note

The data can be accessed via this link: https://osf.io/sjt9p/.

## Ethics Statement

The study was conducted with approval by the Ethical Committee of the Faculty of Behavioural and Movement Sciences of the Vrije Universiteit Amsterdam and all rules, regulations, and guidelines were followed. All subjects gave written informed consent in accordance with the Declaration of Helsinki.

## Author Contributions

KA, AVB, JS, and MD designed the experiments. KA performed the experiments, and analyzed the results. KA, AVB, JS, and MD wrote the manuscript.

## Conflict of Interest Statement

The authors declare that the research was conducted in the absence of any commercial or financial relationships that could be construed as a potential conflict of interest.
